# Cytokeratin profile supports developmental origin of cerebellopontine angle epidermoid cysts

**DOI:** 10.1016/j.bas.2026.106146

**Published:** 2026-06-23

**Authors:** Anselmi Kovalainen, Olli Tynninen, Justiina Huhtakangas, Martin Lehecka

**Affiliations:** a Department of Neurosurgery, Helsinki University Hospital and University of Helsinki, Helsinki, Uusimaa, Finland; b Department of Pathology, Helsinki University Hospital and University of Helsinki, Helsinki, Uusimaa, Finland

**Keywords:** Cerebellopontine angle, Epidermoid cysts, Cytokeratins, Epidermoid carcinoma, Immunohistochemistry

## Abstract

**Introduction:**

Epidermoid cysts (EC) of the cerebellopontine angle (CPA) are thought to arise from ectopic ectodermal cells trapped during the neural tube closure. CPA ECs are sometimes referred to as cholesteatomas, which have an identical histological structure to CPA EC. Reoperations are frequent and malignant transformation can occur. Cytokeratins (CK) are structural proteins of epithelial cell cytoskeleton that may be used to determine developmental lineages of epithelia.

**Research question:**

To explore the epithelial origin of CPA ECs, we analyzed their CK expression patterns.

**Material and methods:**

We analyzed 26 histological samples, including two malignant cases, from 23 patients operated for CPA EC using immunohistochemical methods. Staining for simple epithelia (CK7, CK8, CK18, CK19, CK20), stratified epithelia (CK14) and cell proliferation marker Ki67 were performed.

**Results:**

CK14 was strongly and consistently expressed in all samples of CPA EC epithelium. Variable CK19 expression was seen in all samples, mainly in the basal layer. Focal expression of CK7 and CK8 was detected in over 50 % of samples. No difference in cytokeratin expression was seen in cases requiring reoperation or with malignant transformation. Ki67 index did not differ in cases requiring reoperation (p = 0.55).

**Discussion and conclusion:**

The cytokeratin profile of CPA ECs corresponds with stratified epithelium of ectodermal origin. CPA EC cytokeratin expression pattern differs from previously reported cytokeratin expression of middle ear cholesteatoma, which suggests a different origin. Cytokeratin profile or Ki67 index were not predictive of consequent reoperation or malignant transformation in this cohort.

## Highlights

• CPA EC cytokeratin profile corresponds with previous theories of congenital origin.
• CK profile or Ki67 index did not predict reoperations or malignant transformation.
• CPA EC differ from middle ear cholesteatoma based on CK profile.

## 1 Introduction

Epidermoid cysts (EC) are benign intracranial tumors that consist of a capsule of stratified squamous epithelium and cyst content of lamellar keratin (Perry et al., 2015). Half of intracranial ECs are located in the cerebellopontine angle (CPA), where they represent the third most common benign tumor after schwannomas and meningiomas (Nagasawa et al., 2011). A third of ECs are located in the sellar region (Nagasawa et al., 2011). Intraparenchymal ECs are rare (Kaido et al., 2003). Ectopic ectodermal cells, from the closure of the neural tube during the 3rd and 5th week of gestation, are widely considered the origin of CPA ECs (Mahoney, 1936). During the clinical course of CPA EC, reoperations due to regrowth are common, and malignant transformations have been reported (Sellier et al., 2022; Zuo et al., 2021). As no inherent squamous epithelium has been identified in the CPA, a congenital maldevelopmental origin from ectopic ectodermal cells is probable. Consequently, primary CPA epidermoid carcinomas are likely a result of malignant transformation of CPA EC (Zuo et al., 2021; Garcia et al., 1981). It has been suggested that complicated ectodermal invaginations during the development of the ear are the reason for the common presentation of EC in the CPA (Toglia et al., 1965). Epidermoid cysts are sometimes interchangeably referred to as cholesteatomas due to their similar histological structure (Schiefer and Link, 2008; Kuo et al., 2015; Kountakis et al., 2000).

Cytokeratins (CK) are structural proteins in the cytoskeleton of epithelial cells (Moll et al., 2008). The cytokeratin gene family consists of 54 distinct genes, and the keratin polypeptides are called by their catalog number (e.g. CK7, CK20) (Moll et al., 1982, 2008). Expression patterns of cytokeratins are characteristic of epithelial cell types and tissues, and they can be used as markers to determine the tissue of origin of undifferentiated and metastatic tumors (Miettinen, 2014; Moll et al., 1992; Ordóñez, 2013; Southgate et al., 1999; Bahrami et al., 2008). In human central nervous system (CNS), cytokeratins are expressed exclusively in choroid plexus epithelial cells (Kasper and Karsten, 1988; Miettinen et al., 1986). Cytokeratin expression has been utilized in differential diagnostics of CNS lesions such as craniopharyngeomas, Rathke cleft cysts, and other types of intracranial cysts with variable results (Xin et al., 2002; Uematsu et al., 1990; Tateyama et al., 2001; Coca et al., 1993; Lach et al., 1993). Knowledge on the immunohistochemical properties and cytokeratin expression of CPA ECs is limited to single cases with malignant progression (Uematsu et al., 1990; Raghunathan et al., 2011; Tamura et al., 2006; Narasimhaiah et al., 2023). In those case reports carcinoma cells were positive for CK7, CK5/6 and pancytokeratin antibodies (Raghunathan et al., 2011; Tamura et al., 2006). Studies with larger cohorts have lacked a comprehensive cytokeratin profile of CPA EC (Yawn et al., 2016; Samii et al., 1996; Hasegawa et al., 2016). We hypothesize that mapping the cytokeratin expression of benign CPA EC might give insight into their origin, analogous to studies of malignant epithelial tumors.

In this study we aimed to investigate the developmental origin of CPA EC by characterizing the expression of various cytokeratin types in their capsule epithelium. We hypothesized that the cytokeratin profile of CPA ECs would be consistent with stratified squamous epithelium of ectodermal origin, supporting the developmental theory of origin from ectopic epithelial remnants.

## 2 Materials and methods

**Fig. 1:**
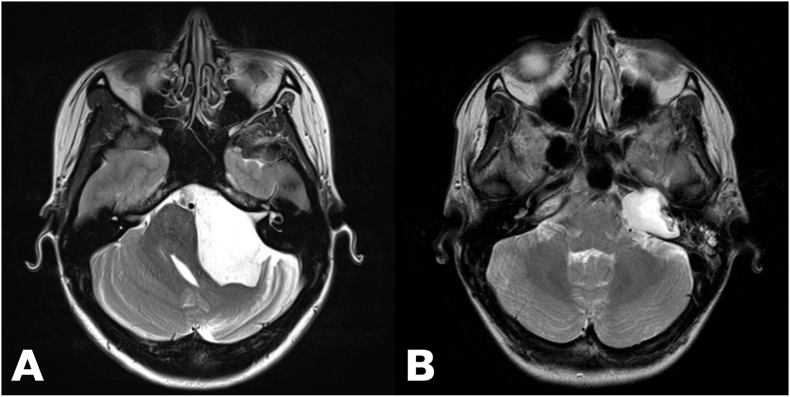
Illustrative cases of cerebellopontine angle epidermoid cysts (CPA EC) included in, and cholesteatoma excluded from the study. A: T2-weighted MRI sequence of left-sided CPA EC which does not invade surrounding bony structures, included in this study. B: T2-weighted MRI sequence of left-sided cholesteatoma involving the petrous part of the temporal bone, excluded from this study.

We identified 31 patients operated for CPA EC between 1975 and 2022 from institutional electronic pathologic databases and surgical logbooks in our previous retrospective study (Kovalainen et al., 2025). The timeframe was selected based on the transition to electronic databases. Of the patients identified, 24 had diagnostic histopathological samples available at a local biobank and were selected to form the final study cohort. The surgeries were performed at a single academic center with a catchment area of 2.2 million (2023). The study was limited to primary CPA epidermoid cysts to study a clinically and anatomically homogenous group. Tumors invading the surrounding bony structures were excluded, as these may represent a different origin (Fig. 1). Surgical approach and strategy were selected according to tumor extension and surgeon preference. Paraffin-embedded histological diagnostic samples were collected from the regional biobank. Consent for use and transfer of samples was obtained in accordance with local legislation. The study plan was approved by the local ethical committee. The study material was handled in accordance with the Declaration of Helsinki. The manuscript was prepared in accordance with the STROBE guideline for cohort studies (Vandenbroucke et al., 2014). All requests for research data require review and approval by the local research committee.

In this study we used a panel of 6 cytokeratin antibodies (CK7, CK8, CK14, CK18, CK19 and CK20) and Ki67 proliferation marker to characterize CPA EC. All antibodies are commonly utilized in diagnostic pathology laboratories. CK14 is typically expressed in stratified epithelia while CK7, CK8, CK18, CK19 and CK20 are cytokeratins of simple (single layer) epithelia (Moll et al., 2008). A focus on cytokeratins of simple epithelia was chosen to clarify the developmental etiology of CPA EC. Immunohistochemical staining was performed using antibodies against cytokeratins CK7 (clone SP52, prediluted antibody, Roche), CK8 (clone C51, dilution 1:200, Zymed), CK14 (clone LL002, dilution 1:100, Invitrogen), CK18 (clone DC 10, dilution 1:100, Dako), CK19 (clone A53-B/A2.26, prediluted antibody, Roche), CK20 (clone SP33, prediluted antibody, Roche) and proliferation antigen Ki67 (clone MIB-1, dilution 1:100). All staining was performed with positive controls. The original histopathological diagnoses were reviewed by a senior neuropathologist (OT). Immunohistochemical stainings were studied by two investigators (AK and OT). Cytokeratin expression of cyst epithelium was analyzed microscopically. A semiquantitative grading scheme was used (negative = less than 5 % positive, weak = 5-25 % positive, moderate = 25-50 % positive, strong = 50-100 % positive). Pattern of cytokeratin expression (superficial vs. basal) was recorded. Proliferation rate of the cyst epithelium (Ki67 index) was evaluated as percentage of positive cells.

The differences in Ki67 index and cytokeratin expression between cases with and without reoperation were evaluated with a nonparametric Mann-Whitney *U* test, with two-tailed exact significance levels reported. Spearman's rank correlation coefficient was used to evaluate associations of continuous or ordinal variables. Statistical analyses were performed using the IBM SPSS Statistics 29.0.2.0 software.

## 3 Results

For the study, 26 histological samples from 23 patients were included (Table 1). One patient was excluded due to incomplete immunohistochemical stainings. Samples of two different operations were analyzed from three patients, including one with malignant transformation. The material included two cases of malignant squamous cell carcinoma; one was malignant at the primary operation; the other one progressed to carcinoma after multiple reoperations at the 4th operation. The study cohort had a male predominance (74 %). In 11 patients the primary surgery was before the year 2000 (48 %). The most common approach was suboccipital retrosigmoid craniotomy (15/23, 65 %). Detailed surgical data from this cohort have been published previously (Kovalainen et al., 2025).

**Table 1:** Study population and descriptive data.

Characteristic	n/Median	%/Range
Patients	23	
Samples analyzed	26	
Age at primary operation, median	35 years	18–56
Craniotomy		
Suboccipital retrosigmoid	15	65 %
Subtemporal	3	13 %
Pterional	1	4 %
Frontotemporal	1	4 %
N/A	3	13 %
Surgeon-assessed extent of resection primary resection		
Gross total	7	30 %
Near total	5	22 %
Subtotal	11	48 %
Imaging-based extent of resection primary resection (n = 19)		
Gross total	4	21 %
Near total	4	21 %
Subtotal	11	58 %
Sex		
Male	17	74 %
Female	6	26 %
Tumor size, median (n = 17)	42 mm	15–52
Follow-up, median	23 years	0.2–48
Reoperation due to regrowth during follow-up	11	48 %
Time from primary operation to first reoperation, median	8.7 years	0.1–24
First sample from operation nmber		
1.	15	65 %
2.	5	22 %
3.	1	4 %
4.	2	9 %

Values are shown as number (%), or median (range) unless indicated otherwise.

If data is not available for whole cohort (n = 23), size of group is indicated in parenthesis.

Extent of resection (EOR) was determined from the primary operation from surgical reports and postoperative imaging separately. EOR was classified as gross total, when both tumor capsule and content was removed, near total when some tumor capsule remained and subtotal when both tumor capsule and content remained.

**Fig. 2:**
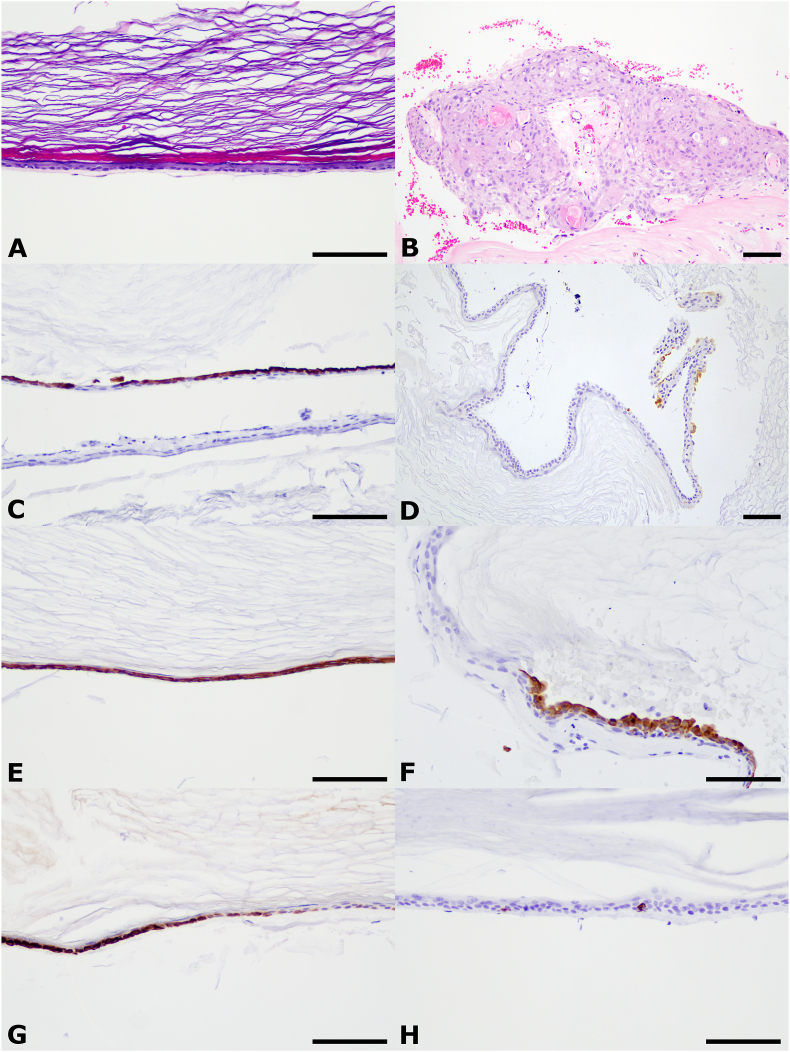
Microscopic images of cerebellopontine angle epidermoid cysts. A: Benign epidermoid cyst with stratified epithelium and lamellar keratin filling. B: Malignant squamous cell carcinoma with thick pleomorphic epithelium. C-H: Immunohistochemical staining for cytokeratins (CK). C: CK7 showed superficial expression in 11 samples (48 %). D: CK8 showed superficial expression in 4 samples (17 %). E: CK14 was uniformly expressed in all samples. F: CK18 showed superficial expression in 2 samples (9 %). G: CK19 showed basal expression in 10 samples (43 %). H: CK20 showed basal expression in 1 sample (4 %). Scale bar = 100 μm.

There was no correlation between expression of any individual cytokeratins and age at operation, preoperative tumor size, or reoperation due to regrowth (Supplementary Table 1). CK14 was most consistently expressed in the EC epithelium (Table 2). CK14 was positive in all 26 EC samples with expression through all the layers of the epithelium (Fig. 2). CK19 showed moderate to strong positivity in 24 samples (92 %) with expression restricted to the basal layer in 10 samples (43 %). Moderate to strong CK7 positivity was detected in 16 samples (62 %) (Fig. 2C). CK7 was expressed exclusively in the superficial layer in 11 samples (48 %). CK20 was predominantly negative with only weak staining detected in 3 samples (12 %). CK8 showed weak to moderate staining in 16 samples (58 %) and 11 samples (42 %) were negative (Fig. 2D).

**Table 2:** Cytokeratin (CK) expression of cerebellopontine angle epidermoid cysts.

Antibody	Negative	%	Weak	%	Moderate	%	Strong	%
CK7	6	26 %	3	13 %	13	57 %	1	4 %
CK8	9	39 %	11	48 %	3	13 %	0	0 %
CK14	0	0 %	0	0 %	0	0 %	23	100 %
CK18	15	65 %	5	21 %	3	13 %	0	0 %
CK19	0	0 %	2	9 %	8	36 %	13	57 %
CK20	23	87 %	3	13 %	0	0 %	0	0 %

First available sample included, n = 23.

Negative = < 5 % positive, Weak = 5-25 % positive, Moderate = 25-50 % positive, Strong = 50-100 % positive.

In malignant tumors the cytokeratin expression pattern did not differ from benign tumors (Table 3).

**Table 3:** Cytokeratin expression after malignant transformation of epidermoid cysts in the current study and literature.

Study	Age	Sex	Previous operations	Pathological diagnosis	Follow-up	Pancytokeratin	CK5/6	CK7	CK8	CK14	CK18	CK19	CK20	Ki67
Raghunathan et al., 2011	60	M	1	Squamous cell carcinoma (sarcomatoid carcinoma)	10 years	++	+++							
Tamura et al., 2006	64	F	1	Squamous cell carcinoma	8 years			+++						48
Current case 1	57	M	0	Epidermoid carcinoma	N/A			++	+	+++	-	+++	-	15
Current case 2	63	M	3	Epidermoid carcinoma	12 years			-	-	+++	-	++	-	30

(−) < 5 % positive, (+) 5–25 % positive, (++) 25–50 % positive, (+++) 50–100 % positive.

The mean Ki67 index of benign ECs was 9 % (SD ± 5) (Supplementary Fig. 1). In the 2 malignant tumors the Ki67 index was higher (15 % and 30 %). There was no statistically significant difference in Ki67 index between benign cases with or without recorded reoperation during follow-up (n = 22, p = 0.55).

## 4 Discussion

In our series the cytokeratin profile of the CPA EC capsule epithelium corresponded to stratified squamous epithelium of ectodermal origin, consistent with prior theories of CPA EC origin, as originally suggested by Boestroem et al., in 1897 (Bostroem, 1897;Mahoney, 1936). The cytokeratin profile was similar in benign ECs and their malignant transformations. The cytokeratin profile or Ki67 proliferation index was not predictive of reoperation or malignant transformation.

### 4.1 Cytokeratins as markers for epithelial tumors

Cytokeratins, also referred to as soft keratins, are structural proteins of the cellular architecture of epithelial cells (Miettinen, 2014). Epithelial cells usually express 4 to 8 distinct cytokeratin types, the number of which may be influenced by inflammation or atrophy (Moll et al., 2008; Ordóñez, 2013). In surgical pathology, immunohistochemistry using cytokeratin antibodies is a fundamental method in diagnosing and typing of epithelial tumors (Chu and Weiss, 2002). The cytokeratin profile of a tumor may also reflect its developmental origin or primary location of a metastatic tumor, and can be used as a serum biomarker to monitor tumor progression (Moll et al., 2008; Chu and Weiss, 2002; Beauchamp et al., 2023). The combination of CK7 and CK20 is one of the most useful immunohistochemical panels for assessing the origin of metastatic cancer (Dum et al., 2022). CK14 is a high molecular weight cytokeratin normally expressed in stratified epithelia such as epidermis, and it is usually co-expressed with CK5 (Moll et al., 2008). Other cytokeratins in our panel (CK7, CK8, CK18, CK19 and CK20) are low molecular weight cytokeratins, which are primarily expressed in simple (one-layered) epithelia such as glandular epithelia in normal human tissues (Moll et al., 2008).

Cytokeratins are in clinical use as serum tumor markers to monitor progression of epithelial cell carcinomas (Barak et al., 2004). While cytokeratins have not been studied as biomarkers for EC, the carbohydrate antigen 19-9 has been suggested as a potential serum biomarker of intracranial EC (Wang et al., 2017). Nevertheless, the role of cytokeratins as biomarkers in EC remains unexplored.

### 4.2 Cytokeratins in epidermoid cysts

Our material supports the proposed ectodermal origin of the stratified squamous epithelium of CPA ECs. We observed strong expression of CK14 in all and at least weak CK19 in all CPA ECs. In normal tissues CK14 expression is a typical feature of stratified squamous epithelium (Broekaert et al., 1990), where it is typically co-expressed together with CK5 (Moll et al., 2008). In intracranial tumors, CK14 expression has been detected in craniopharyngioma (Tateyama et al., 2001). CK19 is normally present in non-keratinizing stratified squamous epithelium, urothelium and also in middle ear mucosa (Olszewska and Sudhoff, 2007). In pathological alterations such as inflammation or dysplasia, expression of CK19 may be induced in epithelial cells that are normally negative for CK19 (Moll et al., 2008). Our study had a higher proportion of males, while there is no clear gender difference in the literature (Shear et al., 2020).

In our material, we detected modest positivity for CK8 and CK18 in some samples. This pair of cytokeratins is typically co-expressed in simple epithelia of parenchymal organs such as liver and pancreas (Moll et al., 2008). CK8 and CK18 may be focally expressed in the basal cell layer of non-keratinizing stratified squamous epithelia which is in line with our observations of CPA EC (Moll et al., 2008). CK8 and CK18 are expressed in most carcinomas, and they stain strongly in adenocarcinomas (Moll et al., 2008). The overall low expression of CK8 and CK18 in CPA EC, including after malignant transformation, further supports their resemblance to normal stratified squamous epithelium without transformation.

Cytokeratin 20 has a very restricted expression pattern in normal tissues. It is expressed in intestinal epithelium, urothelium and neuroendocrine Merkel cells of the skin (Moll et al., 2008). In tumor pathology, CK20 is a potent immunohistochemical marker particularly in combination with CK7 when assessing the origin of metastatic cancer (Dum et al., 2022). We did not detect significant CK20 expression in CPA EC. CK7 expression is sparse or absent in normal stratified squamous epithelia (Moll et al., 2008; Dum et al., 2022). We observed variable CK7 expression in 74 % of CPA EC which may reflect a reaction of EC epithelium to the intracranial environment.

### 4.3 CPA epidermoid cyst vs. cholesteatoma

Our results suggest that CPA ECs and middle ear cholesteatomas have different pathogenesis. CPA ECs are sometimes referred to as (congenital) “cholesteatoma” in clinical practice and medical literature (Nagasawa et al., 2011; Schiefer and Link, 2008; Kuo et al., 2015; Kountakis et al., 2000). Interestingly, the previously reported cytokeratin profile of middle-ear cholesteatoma differs from our findings. Olszewska et al. (Olszewska and Sudhoff, 2007; Olszewska et al., 2005) reported that cholesteatomas of the middle ear showed only weak expression of CK14 and were totally negative for CK19 while van Blitterswijk et al. (Van et al., 1989) reported only weak expression of CK19. Ergün et al. (Ergun et al., 1994) found variable CK14 positivity in middle ear cholesteatoma.

Histopathologically both EC and cholesteatoma are composed of a thin capsule of stratified squamous epithelium and pearly keratin content (Kuo et al., 2015). Unlike CPA EC, cholesteatomas are associated with temporal bone and middle ear component destruction (Nagasawa et al., 2011; Liu et al., 2003; Moriyama et al., 1984). True cholesteatomas arise in the middle ear, and they can be further classified as congenital or acquired, the latter of which may result from a chronic middle ear infection (Kuo et al., 2015; Maniu et al., 2014). Experimentally, in vitro CPA EC cells exhibit similar unique migratory properties as acquired cholesteatomas arising from tympanic membrane suggesting their common origin from the embryonic first branchial groove system (Kountakis et al., 2000). However, our observations on differences in cytokeratin expression between CPA EC and cholesteatoma support their separate pathogenesis.

### 4.4 Malignant transformation

Our material included 2 malignant CPA EC cases. There was no clear difference in cytokeratin profile between benign and malignant EC in our study material suggesting that it carries no prognostic information in CPA EC. No factors predicting malignant transformation were discovered. We observed high Ki67 index in malignant EC as expected from the literature (Tamura et al., 2006; Narasimhaiah et al., 2023). Ki67 can be used as a marker for cell division in both clinical and research use (Ergun et al., 1994).

### 4.5 Limitations

Our study has some weaknesses. A limited number of cytokeratin antibodies were included in our study. Our panel included antibodies for cytokeratins known to be expressed in both stratified and simple epithelia, and was selected to investigate the developmental origin of CPA EC. Therefore, it does not represent a comprehensive analysis of all cytokeratins expressed in CPA EC epithelium. The origin of human cells and tissues can be studied by characterizing their proteins, RNA expression or epigenomic signature (Ye and Sarkar, 2018). Our study cohort is limited to formalin-fixed surgical samples which allow reliable studies of protein expression by immunohistochemical methods. We selected a panel of antibodies that are utilized in routine pathological diagnostics and show reliable results in paraffin sections. While our study sample size is limited for statistical analysis, we consider it sufficient to make conclusions on the cytokeratin profile, especially given the rarity of CPA EC. Furthermore, statistical analyses of differences between benign and malignant CPA EC cytokeratin profile are not feasible due to the rarity of malignant transformation. The surgical samples included in our study have been collected over a long period of time, during which developments in surgical techniques and variation in surgical strategies have occurred. The long inclusion period may have influenced the observed reoperation rate, as postoperative regrowth of CPA EC is presumed to arise from residual capsule epithelium that may be difficult to identify intraoperatively or on postoperative imaging (Kovalainen et al., 2025). Our study focuses on CPA EC to understand the origin of EC in a single anatomical location. Therefore, our results may not be transferable to ECs of other locations. In further studies, a cytokeratin profile of EC located at other sites could give insight to their origin, and compared to our findings of CPA EC.

## 5 Conclusions

The stratified epithelium of CPA EC capsule has an ectodermal cytokeratin profile, consistent with previous theories. Cytokeratin profile or Ki67 index are not predictive of consequent regrowth or malignant transformation in our material. Based on their cytokeratin expression pattern, CPA ECs and middle ear cholesteatomas are likely different entities with different pathogenesis. Further studies on cytokeratin profile of EC of other anatomical locations may provide additional insight into their respective developmental origin.

## Declaration of competing interest

The authors declare that they have no known competing financial interests or personal relationships that could have appeared to influence the work reported in this paper.
