# Supplementary material for: Cytokeratin profile supports developmental origin of cerebellopontine angle epidermoid cysts
Source: Brain Spine. 2026 Jun 23;6:106146. doi: 10.1016/j.bas.2026.106146 (PMC13355422; doi:10.1016/j.bas.2026.106146)
Supplement: Multimedia component 1 [file mmc1.docx]

**Supplementary files**

Supplementary figure 1. Comparison of the distribution of Ki67 index in benign and malignant CPA EC.

**
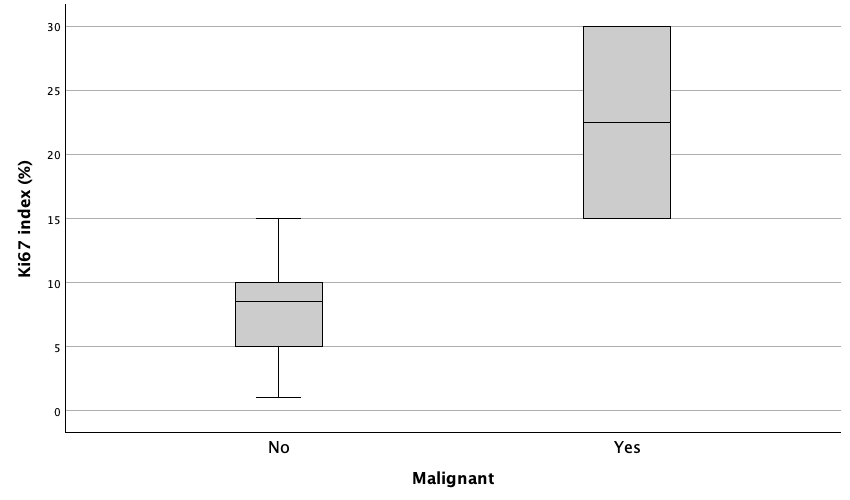
**

Supplementary table 1. Statistical tests

| Variable | Marker | Test used | Test Statistic | p-value |
| --- | --- | --- | --- | --- |
| Any reoperation | Ki67 index | Mann-Whitney U | 9.0 | 0.29 |
| Time to reoperation | Ki67 index | Spearman’s rho | –0.38 | 0.25 |
| Age at primary operation | Ki67 index | Spearman’s rho | –0.09 | 0.68 |
| Tumor size at primary operation | Ki67 index | Spearman’s rho | 0.10 | 0.69 |
| Any reoperation | CK7 | Mann-Whitney U | 17 | 0.82 |
| Time to reoperation | CK7 | Spearman’s rho | 0.21 | 0.54 |
| Age at primary operation | CK7 | Spearman’s rho | 0.28 | 0.20 |
| Tumor size at primary operation | CK7 | Spearman’s rho | –0.91 | 0.73 |
| Any reoperation | CK8 | Mann-Whitney U | 17 | 0.89 |
| Time to reoperation | CK8 | Spearman’s rho | 0.09 | 0.80 |
| Age at primary operation | CK8 | Spearman’s rho | 0.04 | 0.85 |
| Tumor size at primary operation | CK8 | Spearman’s rho | 0.001 | 0.99 |
| Any reoperation | CK14 | Mann-Whitney U | 21 | 1.0 |
| Time to reoperation | CK14 | Spearman’s rho | N/A | N/A |
| Age at primary operation | CK14 | Spearman’s rho | N/A | N/A |
| Tumor size at primary operation | CK14 | Spearman’s rho | N/A | N/A |
| Any reoperation | CK18 | Mann-Whitney U | 13 | 0.53 |
| Time to reoperation | CK18 | Spearman’s rho | –0.16 | 0.65 |
| Age at primary operation | CK18 | Spearman’s rho | 0.01 | 0.97 |
| Tumor size at primary operation | CK18 | Spearman’s rho | –0.31 | 0.23 |
| Any reoperation | CK19 | Mann-Whitney U | 16 | 0.57 |
| Time to reoperation | CK19 | Spearman’s rho | –0.03 | 0.93 |
| Age at primary operation | CK19 | Spearman’s rho | 0.002 | 0.99 |
| Tumor size at primary operation | CK19 | Spearman’s rho | –0.20 | 0.44 |
| Any reoperation | CK20 | Mann-Whitney U | 18 | 1.0 |
| Time to reoperation | CK20 | Spearman’s rho | –0.30 | 0.93 |
| Age at primary operation | CK20 | Spearman’s rho | –0.21 | 0.33 |
| Tumor size at primary operation | CK20 | Spearman’s rho | –0.19 | 0.47 |
